# The effect of insulin on response to intravitreal anti-VEGF injection in diabetic macular edema in type 2 diabetes mellitus

**DOI:** 10.1186/s12886-022-02325-x

**Published:** 2022-02-28

**Authors:** Rajya L. Gurung, Liesel M. FitzGerald, Ebony Liu, Bennet J. McComish, Georgia Kaidonis, Bronwyn Ridge, Alex W. Hewitt, Brendan JT. Vote, Nitin Verma, Jamie E. Craig, Kathryn P. Burdon

**Affiliations:** 1 Menzies Institute for Medical Research, University of Tasmania, 17 Liverpool Street (Private Bag 23), Hobart, Tas 7000 Australia; 2 Department of Ophthalmology, Flinders Health and Medical Research Institute, Flinders University, Adelaide, South Australia; 3 School of Medicine, University of Tasmania, Hobart, TAS Australia

**Keywords:** Diabetic macular edema, Anti-VEGF, Insulin, Visual acuity, Central macular thickness

## Abstract

**Objectives:**

To assess whether insulin therapy impacts the effectiveness of anti-vascular endothelial growth factor (anti-VEGF) injection for the treatment of diabetic macular edema (DME) in type 2 diabetes mellitus.

**Methods:**

This was a retrospective multi-center analysis. The best-corrected visual acuity (BCVA) at 12 months, BCVA change, central macular thickness (CMT), CMT change, and cumulative injection number were compared between the insulin and the oral hypoglycemic agent (OHA) groups.

**Results:**

The mean final BCVA and CMT improved in both the insulin (*N* = 137; *p* < 0.001; *p* < 0.001, respectively) and the OHA group (*N* = 61; *p* = 0.199; *p* < 0.001, respectively). The two treatment groups were comparable for final BCVA (*p* = 0.263), BCVA change (*p* = 0.184), final CMT (*p* = 0.741), CMT change (*p* = 0.458), and the cumulative injections received (*p* = 0.594). The results were comparable between the two groups when stratified by baseline vision (*p* > 0.05) and baseline HbA1c (*p* > 0.05).

**Conclusion:**

Insulin therapy does not alter treatment outcomes for anti-VEGF therapy in DME.

**Supplementary Information:**

The online version contains supplementary material available at 10.1186/s12886-022-02325-x.

## Introduction

Diabetic macular edema (DME) is the most common cause of vision loss in both type 1 (T1) and type 2 (T2) diabetes mellitus (DM). The reported incidence of DME is highly variable ranging from 0 to 3% in newly diagnosed cases to 28–29% in chronic diabetic patients with over 20 years disease duration [1]. Similarly, the reported prevalence of DME ranges widely from 0.8–7.5% [2, 3]. The pathogenesis of DME is complex and poorly understood. Of the various risk factors, poor glycaemic control is well proven to increase the risk of DME [4, 5] and a large body of evidence supports the long-term beneficial effects of tight glycemic control in diabetic patients [6]. Consequently, insulin is one of the most widely used hypoglycemic agents in all forms of DM [7, 8], with the benefit of improving glycemic control in addition to lowering rates of diabetes-related complications. However, insulin use can have unintended effects, with reports of increased cardiovascular events and increased colorectal cancer in patients on insulin therapy [9, 10]. There are also several reports of increased risk of DME with the use of insulin therapy in diabetics [11]. Intensive insulin therapy was first linked to retinopathy in the 1980s in T1 diabetes patients [12]. Since then, there have been similar reports of worsening of diabetic retinopathy (DR), including DME, in T2 diabetic patients taking insulin therapy for glycemic control [13, 14]. A study by Zapata et al. showed an increased risk of diffuse DME in T2 diabetics on insulin therapy (odds ratio = 1.4, *p* = 0.036) [15]. Additionally, a meta-analysis of 14 studies showed insulin treatment to be associated with increased risk of DME (relative risk = 3.416; 95%CI = 2.42, 4.83) [11]. The higher risk of DR progression amongst the insulin group may partly be attributed to disease severity, and DR continues to progress despite intensive insulin therapy [16, 17]. In these patients, the association with insulin treatment might simply reflect the severity of the underlying disease rather than the adverse effect of insulin treatment. Nevertheless, other studies have demonstrated insulin therapy to be an independent risk factor for DR and DME [15, 18]. Further, a meta-analysis by Zhao et al. clearly showed that the association of insulin with DME risk is independent of baseline glycosylated hemoglobin (HbA1c) levels [19].

Various hypotheses have been proposed to explain the adverse effect of insulin therapy, though the exact underlying molecular mechanisms remain elusive. Insulin acts as a growth factor and it is hypothesized to worsen DR severity by promoting 1) endothelial cell proliferation [20], 2) loss of basement membrane and surrounding pericytes [21], and 3) synthesis of pro-angiogenic factors like VEGF [22]. Insulin increases the expression of VEGF receptors and reactive oxygen species, which in turn triggers retinal neovascularization and DME. In support of this hypothesis, rapid glycemic control in diabetic rat models has shown increased expression of VEGF mRNA and protein levels mediated by hypoxia‐inducible factor‐1α [23]. Similarly, another study found increased vascular leakage in mice treated with insulin, apparently involving betacellulin mediated epidermal growth factor signaling pathway [24]. A study of T2 diabetic patients by Henricsson et al. [13] found elevated levels of IGF-1, a protein that can decrease blood glucose levels, in patients who had worsening of DR three years after insulin initiation.

Thus, the association of insulin with increased risk and severity of DME may have an impact on response to intraocular anti-VEGF therapy, the latest standard of care for DME. To date, there are very few studies investigating the possible influence of insulin therapy on response to anti-VEGF injection [25, 26]. The aim of the present study was, therefore, to assess whether insulin therapy in T2DM patients impacts DME response to anti-VEGF therapy compared with using oral hypoglycemic agents (OHA).

## Methodology

### Study design

This was a retrospective multi-center study. Participants were selected from one of two studies, the Tasmanian Ophthalmic Biobank (University of Tasmania) or the Genetic Risk Factors in Complications of Diabetes (Flinders University). The Tasmanian Ophthalmic Biobank is a collaboration between the University of Tasmania and Tasmanian eye clinics established to collect clinical information and DNA samples from residents of Tasmania with a variety of ocular diagnoses. For enrolment, participants must have had a recent ophthalmic examination and be over 18 years of age. The Genetic Risk Factors in Complications of Diabetes is based at Flinders University (Adelaide, Australia) and includes patients over the age of 18 years with a diagnosis of T1 diabetes or medically treated T2 diabetes.

### Participants

Patients, who commenced any intravitreal anti-VEGF injections (Aflibercept, Regeneron; Bevacizumab, Genentech; Ranibizumab, Novartis;) between 2013 and 2019 for the treatment of DME secondary to T2 diabetes were identified from both cohorts for inclusion in this study. DME cases were defined as those with clinically diagnosed center-involving DME and confirmed by central macular thickness (CMT) ≥ 315 microns as measured by spectral-domain optical coherence tomography (SD-OCT; Heidelberg Spectralis; Heidelberg Engineering Inc., Heidelberg, Germany). Eyes with cysts in the central 1000 microns were also included in this study, independent of the CMT parameter. This study excluded patients who had undergone vitreoretinal surgery or had received any systemic anti-VEGF therapy or intra-ocular steroid in the six months preceding the initiation of anti-VEGF injection. Further, DME patients with severe media opacity obstructing clear visualization of the macula, and/or with incomplete follow-up data were also excluded from the study. All treatment decisions, including the type of anti-VEGF injection, the treatment, and the re-treatment criteria were based at the discretion of the treating physician. The better responding eye was included as the study eye for patients receiving bilateral anti-VEGF injections.

### Clinical data collection

Data for 12 months after the date of the first injection was collected retrospectively from the electronic database. The data included clinical and demographic characteristics: age, sex, lipid profile, hypertensive status, diabetic nephropathy status, duration of diabetes, baseline glycemic control (HbA1c), smoking status, best-corrected visual acuity (BCVA), CMT, intraocular pressure (IOP), laterality of the injected eye, lens status, duration of retinopathy, severity of retinopathy, pan-retinal photocoagulation (PRP) at baseline, anti-VEGF injections (number and type) and adverse drug events during/post-injection. Both proliferative diabetic retinopathy and severe non-proliferative diabetic retinopathy were combined as the severe DR group. Hypertension was defined as systolic BP level of ≥ 140 mmHg and/or diastolic BP level of ≥ 90 mmHg or any participants on antihypertensive medications. Likewise, hyperlipidemia was defined as total cholesterol greater than or equal to 4 mmol/L, or current use of lipid-lowering medication. Nephropathy was defined as the presence of microalbuminuria (30–300 mg/d) or macroalbuminuria (> 300 mg/d) or patients undergoing dialysis or who had received renal transplantation. Diabetes treatment categories were defined as OHA group vs insulin group. The insulin group comprised of participants taking only insulin or insulin in combination with other OHAs and, the OHA group comprised of participants taking only oral medication(s) for diabetic control. The date/year of insulin therapy initiation, formulation of insulin therapy, and the types of OHA were also noted if data were available. For statistical analysis, Snellen’s best-corrected visual acuity (BCVA) was converted to approximate early treatment diabetic retinopathy study (approxETDRS) letter scores [27].

### Outcome measures

The primary outcome was final BCVA at 12 months after the first intravitreal anti-VEGF injection. Secondary outcomes included final CMT and cumulative number of injections over 12 months. These outcomes were compared between the insulin and OHA treatment groups. The BCVA change and CMT change at the end of 12 months were also compared between the two groups.

To explore the effects of likely confounding factors, we also stratified the participants based on baseline vision (< 70 approxETDRS vs ≥ 70 approxETDRS) and HbA1c level (≤ 7.0 g/dl vs > 7.0 g/dl). We then explored difference in outcomes when limiting the cohort to those with good final vision (vs ≥ 70 approxETDRS) to ensure the effects were not due to greater variability in visual acuity measures in patients with poor vision.

### Statistical analysis

Statistical analyses were performed using R version 4.0.2 (http://www.R-project.org/). Descriptive statistics included the mean with standard deviation (SD) and median (minimum–maximum) for numerical variables. After assessing the normality of all quantitative variables by visualizing the Q-Q plot and histogram outputs, parametric or non-parametric tests were applied where applicable. Paired t-test was used to compare final vision and final CMT with baseline values in each treatment group (insulin and OHA group). Between-group analyses of the two treatment groups were undertaken using the independent t-test or the Mann–Whitney U test for continuous variables and the Chi-square test for categorical variables. Sex (male:female), hyperlipidemia (yes:no), hypertension (yes:no), nephropathy (yes:no), smoking status (yes:no), severe DR (yes:no), PRP at baseline (yes:no), laterality of eye (R/L) and lens status (phakic:pseudophakic) were dichotomized for statistical analyses. A multivariable linear regression analysis was conducted to account for any confounding effect of the baseline characteristics. Clinically relevant variables from previous studies and those significant in the univariable analyses were selected for the multivariable regression. Tests were considered significant at *p* < 0.05.

## Results

A total of 255 diabetic patients receiving anti-VEGF injections were identified. Of these, 35 were T1DM patients and were excluded from the study. Of the remaining 220 T2 patients, 198 met the inclusion criteria. The baseline and clinical characteristics stratified by treatment (insulin vs OHA) are summarized in Table 1. There were 137 patients in the insulin group and 61 in the OHA group. The two groups were comparable in their baseline BCVA, baseline CMT, age, sex, duration of DR, BMI, laterality of eye, hyperlipidemic and smoker status, and the type of anti-VEGF injections received. However, the insulin group had a significantly longer duration of DM (*p* < 0.001), more severe grade of DR (*p* = 0.028), poorer DM control (*p* = 0.033), a higher proportion of hypertensive patients (*p* = 0.014), and a greater proportion of nephropathy (*p* = 0.002) compared to the OHA group. Likewise, more patients in the insulin group had received PRP laser therapy (*p* = 0.002), whereas more patients had undergone cataract surgery (*p* = 0.019) in the OHA group. Bevacizumab was the chosen anti-VEGF agent in more than half the patients in each treatment group (insulin = 54.70%; OHA = 55.70%).

**Table 1:** Baseline and clinical characteristics of patients in each treatment group

Variables	Insulin (*N* = 137)	OHA (*N* = 61)	*P* value
Baseline BCVA (approxETDRS letters)	64.08 (12.99)	64.07 (12.60)	0.994
Baseline CMT (microns)	380.33 (101.54)	396.64 (113.77)	0.316
Lens status (% Pseudophakic)	62.80	78.70	**0.019**
Age (years)	68.19 (10.12)	69.43 (10.34)	0.431
Male (%)	64	31	0.497
Laterality of eye (% RE)	46.70	50.80	0.352
Hypertension (% positive)	89.10	75.40	**0.014**
Hyperlipidemia (% positive)	89.80	86.90	0.354
Nephropathy (% positive)	64.20	41.00	**0.002**
Smoker (%)	48.90	52.50	0.379
BMI (%)	34.38 (7.50)	32.47 (8.08)	0.108
Diabetes duration (years)	22.41 (8.17)	17.95 (9.55)	** < 0.001**
HbA1c (mg/dl)	8.66 (1.71)	7.68 (1.21)	**0.033**
PRP at baseline (% positive)	47.40	24.60	**0.002**
DR duration (years)	7.74 (4.06)	7.62 (3.42)	0.848
Severe DR (%)	51.80	36.10	**0.028**
*Injection type*			0.599
Bevacizumab (%)	54.70	55.70	
Ranibizumab (%)	17.50	23.00	
Aflibercept (%)	11.70	11.50	
Mixed (%)	16.10	9.80	

*Abbreviations*: *BCVA* Best corrected visual acuity, *BMI* Body-mass index, *CMT* Central macular thickness, *DR* Diabetic retinopathy, *approxETDRS* Approximate early treatment diabetic retinopathy study, *OHA* Oral hypoglycemic agent, *PRP* Pan-retinal photocoagulation, *RE* Right-eye. Data are presented as means (SD) for continuous variables and percentage (%) for categorical variables. Independent t-test was conducted for continuous variables and Chi-square test for categorical variables. Significant *p*-values are in bold

### Outcome measures according to diabetes treatment type

**Fig. 1:**
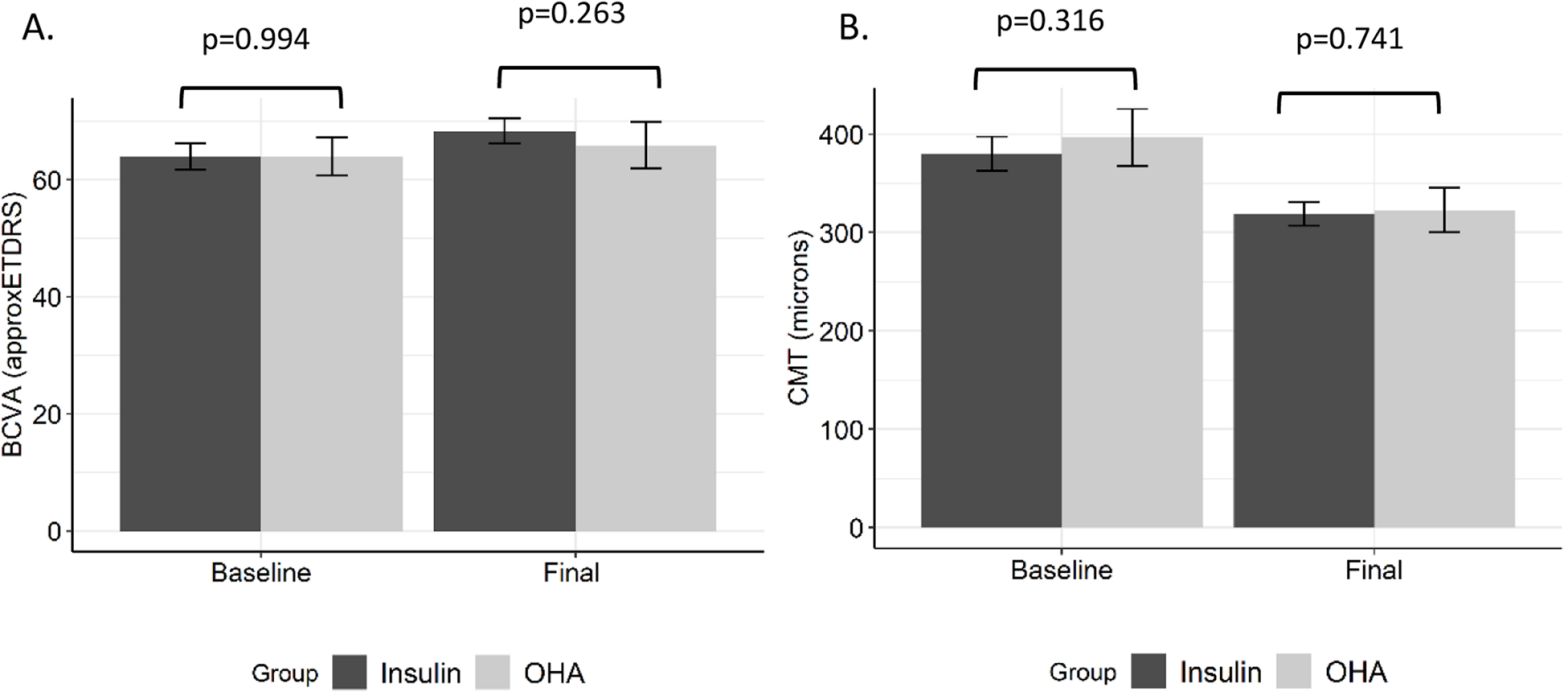
Best-corrected visual acuity and central macular thickness outcome at baseline and the end of 12 months (final) of anti-VEGF therapy by type of diabetes treatment. BCVA: best-corrected visual acuity; approxETDRS: approximate early treatment diabetic retinopathy study; CMT: central macular thickness; OHA: oral hypoglycemic agent; error bars represent 95% confidence interval for mean;* p* value is for comparing the BCVA and CMT between the treatment groups at each visit

After 12 months of anti-VEGF treatment, the mean final BCVA (68.33 ± 12.68 approxETDRS letters) improved significantly from baseline (64.08 ± 12.99 approxETDRS letters) in the insulin group (*p* < 0.001, 95%CI = 2.34, 6.14). In the OHA group, the final BCVA (65.98 ± 15.44 approxETDRS letters) increased from baseline (64.07 ± 12.60 approxETDRS letters), although the improvement was not statistically significant (p = 0.199, 95%CI = -1.04, 4.87). The two groups had similar final BCVA at the end of 12 months (*p* = 0.263, 95%CI = -1.77, 6.46), (Fig. 1).

Furthermore, the final CMT (insulin = 318.90 ± 68.86 microns; OHA = 322.74 ± 88.55 microns) was significantly reduced from baseline (insulin = 380.33 ± 101.54 microns; OHA = 396.64 ± 113.77 microns) in both treatment groups, (insulin = *p* < 0.001, 95%CI = -78.68, -44.17; OHA = *p* < 0.001, 95%CI = -105.37, -42.42). However, there was no significant difference in final CMT between the treatment groups, (*p* = 0.741, 95%CI = -26.74, 19.06), (Fig. 1). Likewise, the two treatment groups received similar number of injections by the end of 12 months, (insulin = 8.25 ± 3.04; OHA = 8 ± 3.26; *p* = 0.594, 95%CI = -0.69, 1.20). The change in vision (insulin = 4.25 ± 11.24; OHA = 1.92 ± 11.55) and CMT (insulin = -61.42 ± 102.13; OHA = -73.90 ± 122.89) was also comparable between the two groups (BCVA change, *p* = 0.184, 95%CI = -1.11, 5.77; CMT change, *p* = 0.458, 95%CI = -20.58, 45.53).

### Outcome measures stratified by baseline vision and DM control

For patients with mean baseline BCVA < 70 approxETDRS letters, there was no significant difference in the final BCVA, BCVA change, CMT outcomes, or cumulative injection number across the two treatment groups, (*p* > 0.05), Table 2. Likewise, the final BCVA, BCVA change, final CMT, CMT change, and number of injections were also comparable across the two groups for the cohort with BCVA ≥ 70 approxETDRS letters (*p* > 0.05), Table 2. Similarly, no significant difference was observed between the two groups when stratified according to DM control (HbA1c > 7.0 or ≤ 7.0 g/dl), with both groups having comparable outcomes across all the parameters (*p* > 0.05), Table 3.

**Table 2:** Anti-VEGF treatment outcome in patients receiving insulin or OHA for DM, stratified by baseline vision

	**Insulin**	**OHA**	*P* value^*^ (95% CI)
**BCVA ≥ 70 approxETDRS letters**	***N* = 75**	***N* = 32**	
Baseline BCVA (approxETDRS letters)	73.53 (3.61)	73.03 (4.02)	0.546 (-1.15, 2.15)
Final BCVA (approxETDRS letters)	73.88 (5.81)	73.66 (6.51)	0.867 (-2.45, 2.89)
BCVA change (approxETDRS letters)	0.35 (6.08)	0.62 (6.99)	0.689 (-4.99, 1.00)
Baseline CMT (microns)	345.84 (58.56)	356.19 (81.10)	0.517 (-42.27, 21.57)
Final CMT (microns)	302.73 (47.29)	316.16 (69.98)	0.326 (-40.68, 13.83)
CMT change (microns)	-43.09 (63.88)	-40.03 (86.81)	0.624 (-32.00, 21.00)
Injection number	8.21 (2.99)	8.03 (3.08)	0.779 (-1.11, 1.47)
**BCVA < 70 approxETDRS letters**	***N* = 62**	***N* = 29**	
Baseline BCVA (approxETDRS letters)	52.65 (10.86)	54.17 (11.36)	0.547 (-6.58, 3.53)
Final BCVA (approxETDRS letters)	61.61 (15.28)	57.52 (17.96)	0.294 (-3.66, 11.85)
BCVA change (approxETDRS letters)	8.97 (13.99)	3.34 (15.10)	0.063 (0.00, 10.00)
Baseline CMT (microns)	422.05 (124.99)	441.28 (128.52)	0.505 (-76.69, 38.24)
Final CMT (microns)	338.45 (84.56)	330 (106.18)	0.708 (-36.26, 91.62)
CMT change (microns)	-83.60 (131.88)	-111.28 (145.80)	0.259 (-26.00, 81.99)
Injection number	8.31 (3.13)	7.97 (3.51)	0.657 (-1.19, 1.87)

*Abbreviations*: *BCVA* Best corrected visual acuity, *CMT* Central macular thickness, *CI* Confidence interval, *DM* Diabetes mellitus, *approxETDRS* Approximate early treatment diabetic retinopathy study, *OHA* Oral hypoglycemic agent

Data are presented as means (SD) for continuous variables and percentage (%) for categorical variables. N represents the total case number in each group. *p-values are for a difference between insulin and OHA group. 95% CI is for the difference between the means of the two groups. Independent t-test/ Mann–Whitney U test for continuous variables between treatment groups; Significant p-values are in bold

**Table 3:** Outcome stratified by baseline HbA1c level

	**Insulin**	**OHA**	*P* value*(95% CI)
**HbA1c > 7.0 g/dl**	***N* = 111**	***N* = 40**	
Baseline BCVA (approxETDRS letters)	64.88 (12.14)	63.05 (14.42)	0.476 (-3.27, 6.94)
Final BCVA (approxETDRS letters)	69.76 (9.51)	65.68 (16.48)	0.145 (-1.46, 9.62)
BCVA change (approxETDRS letters)	4.87 (10.11)	2.63 (12.75)	0.434 (-1.00, 5.00)
Baseline CMT (microns)	386.16 (109.14)	393.25 (119.46)	0.743 (-50.12, 35.95)
Final CMT (microns)	319.37 (69.67)	315.05 (84.72)	0.774 (-25.57, 34.21)
CMT change (microns)	-66.78 (107.51)	-78.20 (123.30)	0.704 (-24.00, 34.99)
Injection number	8.27 (3.08)	7.60 (3.22)	0.259 (-0.50, 1.84)
**HbA1c ≤ 7.0 g/dl**	***N = 26***	***N = 21***	
Baseline BCVA (approxETDRS letters)	60.65 (15.95)	66.00 (8.06)	0.145 (-12.61, 1.91)
Final BCVA (approxETDRS letters)	62.23 (20.70)	66.57 (13.60)	0.393 (-14.48, 5.80)
BCVA change (approxETDRS letters)	1.58 (15.14)	0.57 (8.97)	0.845 (-5.00, 5.00)
Baseline CMT (microns)	355.42 (53.72)	403.10 (104.55)	0.068 (-99.11, 3.77)
Final CMT (microns)	316.88 (66.58)	337.38 (95.82)	0.411 (-70.57, 29.58)
CMT change (microns)	-38.54 (72.18)	-65.71 (124.70)	0.391 (-26.99, 89.99)
Injection number	8.19 (2.91)	8.76 (3.28)	0.538 (-2.42, 1.28)

*Abbreviations*: *BCVA* Best corrected visual acuity, *CMT* Central macular thickness, *CI* Confidence interval, *approxETDRS* Approximate early treatment diabetic retinopathy study, *OHA* Oral hypoglycemic agent

Data are presented as means ± SD. N represents the total case number (percentage) in each group. *p-values are for a difference between insulin and OHA group. 95% CI is for the difference between the means of the two groups. Independent t-test/Mann–Whitney U test for continuous variables between treatment groups; Significant p-values are in bold

As several measures were significantly different between the two treatment groups at baseline (Table 1), a multivariable regression analysis was done to look at the possible confounding effect of these baseline parameters on the final BCVA (Additional File: Supplementary S1). However, even after adjusting for all the potential confounders, the final BCVA was found to be comparable between the insulin and OHA groups.

When stratified by injection type (bevacizumab and ranibizumab), the insulin and OHA groups showed comparable final visual and CMT outcomes after 12 months, (Additional File: Supplementary S2). A separate analysis for “aflibercept” and “mixed injection” was not done owing to the small cohort sizes (Table 1).

A further sub-analysis including only patients with good outcome at the end of 12 months i.e. final BCVA ≥ 70 approxETDRS (*N* = 120; Insulin = 86, OHA = 34) also did not show any significant difference in the visual and CMT outcome between the two treatment groups, (Additional File: Supplementary S3).

## Discussion

In our study, patients in both the insulin and OHA groups had comparable visual and anatomical outcomes in response to anti-VEGF therapy for the treatment of DME. There was no significant difference between the groups in terms of final BCVA, final CMT, BCVA/CMT change, and cumulative injection number. However, the insulin group had worse DM control at presentation, reflected by a significantly higher baseline HbA1c compared to the OHA group. We therefore conducted a sub-analysis stratified by baseline HbA1c to test for any influence of baseline DM control on the outcome, however, no significant difference was observed. Similar results were obtained in a sub-analysis stratified by baseline vision. Around half the patients in our study had baseline vision ≥ 70 approxETDRS letters, which remained stable regardless of DM treatment type (Table 2). Previous studies have shown improved final vision in patients with good baseline vision, although improvements are typically less than in those with poor baseline vision [28]. Conversely, there have also been reports of poor final vision in patients with poor starting vision [28, 29]. In our study, for both categories of baseline vision, the two treatment groups had comparable final BCVA and CMT, indicating similar effectiveness of anti-VEGF therapy, regardless of diabetes treatment modality or baseline vision. Moreover, no significant difference was seen between the two treatment groups even in the sub-cohort with a good visual outcome at the end of 12 months, (Final BCVA ≥ 70approxETDRS). Several potential confounders were present in this study; the insulin group had a significantly longer duration of DM as well as higher proportions of severe retinopathy and higher proportions of PRP laser at baseline. Likewise, the proportions of nephropathy, HTN as well as phakic participants, were significantly higher in the insulin group. However, vision and CMT at 12 months had improved significantly in this group, indicating no adverse effects of insulin on treatment outcome. Further, a multivariable regression analysis adjusting for the baseline parameters showed no difference in the final outcome between the two treatment groups (Additional File: Supplementary S1). The previously reported association of insulin with the progression of DR/DME might be due to pre-existing poor diabetes control before the initiation of insulin therapy, possibly owing to the hyperglycemic memory phenomenon [30]. In addition, previous frontline treatment for DME (grid/focal laser) [31] was less effective than current anti-VEGF treatments and DME typically progressed in most patients [32]. Our study shows that insulin treatment per se does not influence anti-VEGF treatment outcomes.

A study by Matsuda et al. [25] analyzed 96 T2DM (49 insulin, 46 OHA) patients receiving anti-VEGF injections for DME. They also found no significant difference in visual outcomes between the two treatment groups at the end of 12 months, though both groups had significant improvement in vision. Another recent study by Logeswaran et al*.* [26] looked at the possible influence of insulin therapy on macular thickness reduction in patients receiving intravitreal anti-VEGF injections. Again, this study failed to identify any significant difference between the insulin and the non-insulin group. However, the study had a very short follow-up duration of only one month and the two patient groups were evaluated only on macular thickness criteria. The current study extended this duration to one more relevant to extended patient treatment regimens and also assessed visual outcome, which is arguably more important to patient function than macular thickness. These real-world findings corroborate those from a randomized clinical trial [33]. A two-year post hoc analysis of the RIDE and RISE trials, exploring the effect of systemic risk factors for DME, showed a similar visual response to the anti-VEGF agent, ranibizumab, between insulin and non-insulin patient groups. The post hoc analysis further classified the treatment group into three sub-groups comprising insulin only, insulin plus other OHAs, and other OHAs.

This study has some limitations that are inherent to a retrospective observational design and a relatively small cohort size. A true effect of insulin on the treatment of DME in T2DM would require comparison between T2DM patients “only on OHA” vs patients “only on insulin (without prior use of OHA)”. However, for the majority of T2DM patients, including those in our study, the initial drug of choice for the management of hyperglycemia is OHA, followed by insulin in the event they fail to respond to OHA or if any side effects occur [34]. Consequently, most, if not all, T2DM patients would commence on OHA, and hence a true treatment naïve cohort for insulin (without prior use of OHA) would not be feasible. Information regarding the time point of insulin therapy initiation and duration of insulin therapy would have been beneficial. This would have allowed exploration of the effect of acute vs chronic insulin therapy on anti-VEGF response. However, considering the fact that both the insulin and OHA group had a mean DM duration of more than 8 years, most of our cases are likely to be chronic insulin users and our study would have missed the early worsening phase of DR/DME post-initiation of insulin treatment. Further, data on the formulations and methods of insulin administration were not available owing to the retrospective design. For similar reasons, detailed data regarding the type of OHAs used were also not available for all the participants, and hence a more in-depth analysis could not be done. Thiazolidinediones (a type of OHA) have been suggested to be associated with DME in a few case reports [35, 36], whereas other OHAs such as dipeptidyl peptidase-4 (DPP4) inhibitors [37] and sodium-glucose cotransporter 2 inhibitors (SGLT2i) [38] have been reported to be protective against DR. However, a large, cross-sectional ACCORD Eye Study [39] demonstrated no association between thiazolidinediones and DME in T2 diabetics and similarly, a recent study by Kang et al. [40] on the add-on effect of DPP4 on DR in T2 diabetics showed inconclusive evidence. Nevertheless, a larger prospective study on T2DM patients receiving anti-VEGF injections, with details on the different insulin formulations, duration of insulin therapy, and various types of OHAs, would provide a better understanding of the possible adverse effects of different treatment modalities on DME outcomes. As outlined in the methodology section, the Snellen’s visual acuity in this study was converted to approxETDRS. Such conversion, however, cannot be assumed to be equivalent to “true” ETDRS letter scores obtained using a standard ETDRS chart, and hence should be interpreted with caution, especially when dealing with few letters difference [27]. Further, we evaluated the effect of glycemic control at baseline but were unable to assess it at final follow-up due to insufficient data. Finally, a combination of different anti-VEGF agents was used over the one-year period for the majority of our participants, reflecting real-world practice. Analysis by injection sub-type showed similar outcomes in the two groups (Additional File: Supplementary S2).

## Conclusion

In summary, our study showed that insulin therapy does not alter visual outcomes for T2 diabetics receiving anti-VEGF injections and patients do not need to alter their diabetes medication to optimize their eye care. Of key clinical relevance, we found no association between insulin treatment and sub-optimal vision or CMT outcome. In fact, these patients showed significant improvements in both of these outcomes. As insulin improves glycemic control, it may even be positively associated with a better anti-VEGF response in the long term. A prospective study with longer follow-up would help to explore this further.

## Supplementary Information

**Additional file 1.**

## Data Availability

Data are available upon reasonable request.
