# Supplementary material for: The effect of insulin on response to intravitreal anti-VEGF injection in diabetic macular edema in type 2 diabetes mellitus
Source: BMC Ophthalmol. 2022 Feb 28;22:94. doi: 10.1186/s12886-022-02325-x (PMC8883612; doi:10.1186/s12886-022-02325-x)
Supplement: Supplementary file 1 — Additional file 1. [file 12886_2022_2325_MOESM1_ESM.docx]

**Additional File**

**Title:**

**The effect of insulin on response to intravitreal anti-VEGF injection in diabetic macular edema in type 2 diabetes mellitus.**

**Authors:** Rajya L Gurung^1^, Liesel M. FitzGerald^1^, Ebony Liu^2^, Bennet J McComish^1^, Georgia Kaidonis^2^, Bronwyn Ridge^2^, Alex W Hewitt^1,3^, Brendan JT Vote^3^, Nitin Verma^3^, Jamie E Craig^2^, Kathryn P Burdon^1^

^1^ Menzies Institute for Medical Research, University of Tasmania, Hobart, TAS, Australia

^2^Department of Ophthalmology, Flinders Health and Medical Research Institute, Flinders University, Adelaide, South Australia

^3^ School of Medicine, University of Tasmania, Hobart, TAS, Australia

**Correspondence:**

Rajya L Gurung,

Menzies Institute for Medical Research, University of Tasmania, 17 Liverpool Street (Private Bag 23), Hobart, Tas, 7000.

Tel: +61 433525558

E-mail: [rajyalaxmi.gurung@utas.edu.au](mailto:rajyalaxmi.gurung@utas.edu.au).

ORCIDid: <https://orcid.org/0000-0003-0816-9823>

| **Supplementary S1.** **Univariable and multivariable linear regression analyses investigating factors predictive of final BCVA** | | | | | | |
| --- | --- | --- | --- | --- | --- | --- |
| **Variables** | **Univariable** | | | **Multivariable** | | |
|  | ***B*** | **95% CI** | **P** | ***B*** | **95% CI** | **P** |
| Drug type (ref=Insulin) | -2.34 | -6.46, 1.77 | 0.264 | -3.37 | -6.80, 0.06 | 0.054 |
| Baseline BCVA  (approxETDRS ) | 0.668 | 0.55, 0.78 | **<0.001** | 0.66 | 0.53, 0.78 | **<0.001** |
| Baseline CMT  (microns) | -0.022 | -0.03, -0.004 | **0.015** | 0.014 | 0.00, 0.02 | 0.064 |
| Injection number | 0.290 | -0.32, 0.90 | 0.352 | - | - | - |
| *Injection type* |  |  |  |  |  |  |
| Aflibecept vs Bevacizumab(ref) | 10.31 | 4.32, 16.30 | **<0.001** | 4.20 | -0.81, 9.21 | 0.09 |
| Ranibizumab vs Bevacizumab(ref) | 5.25 | 0.34, 10.17 | **0.036** | 2.11 | -1.95, 6.19 | 0.306 |
| Mixed vs Bevacizumab(ref) | 4.44 | -1.08, 9.97 | 0.114 | 2.78 | -1.68, 7.25 | 0.219 |
| Ranibizumab vs Aflibercept(ref) | -5.06 | -11.95,1.83 | 0.149 | -2.08 | -7.72, 3.55 | 0.467 |
| Mixed vs Aflibercept(ref) | -5.87 | -13.21, 1.46 | 0.116 | -1.41 | -7.57, 4.74 | 0.651 |
| Mixed vs Ranibizumab(ref) | -0.81 | -7.31, 5.68 | 0.805 | 0.66 | -4.47, 5.51 | 0.797 |
| DR duration | -0.64 | -1.13, -0.16 | **0.009** | -0.25 | -0.68, 0.16 | 0.233 |
| DR severity (Severe=ref) | -2.83 | -6.63, 0.96 | 0.143 | - | - | - |
| PRP (No=ref) | -4.03 | -7.88, -0.18 | **0.040** | -2.17 | -5.39, 1.04 | 0.183 |
| Lens status  (phakic=ref) | -5.25 | -9.27, -1.24 | **0.010** | -3.77 | -7.16, -0.38 | **0.029** |
| Age (years) | -0.19 | -0.38, -0.01 | **0.038** | -0.10 | -0.27, 0.06 | 0.223 |
| Sex (Female=ref) | 2.76 | -1.17, 6.70 | 0.169 | - | - | - |
| Smoker (No=ref) | -1.17 | -4.98, 2.64 | 0.546 | - | - | - |
| Nephropathy (No=ref) | -0.81 | -4.67, 3.04 | 0.678 | -0.24 | -3.43, 2.94 | 0.879 |
| Hyperlipidemia  (No=ref) | -0.39 | -6.46, 5.68 | 0.899 | - | - | - |
| HTN (No=ref) | 1.65 | -3.66, 6.97 | 0.540 | - | - | - |
| BMI (kg/m^2^) | 0.03 | -0.21, 0.28 | 0.797 | -0.08 | -0.28, 0.11 | 0.384 |
| DM duration (years) | -0.14 | -0.35, 0.07 | 0.189 | - | - | - |
| HbA1c | 0.93 | -0.22, 2.09 | 0.115 | 0.36 | -0.66, 1.38 | 0.489 |
| Abbreviations: BCVA=best corrected visual acuity; BMI=body mass index; CI=confidence interval; CMT=central macular thickness; DME=diabetic macular edema; DM=diabetes mellitus; DR=diabetic retinopathy; approxETDRS=approximate early treatment diabetic retinopathy study; HTN=hypertension; PRP=pan-retinal photocoagulation; ref=reference  Multivariable: adjusted for drug type, baseline BCVA, baseline CMT, injection type, DR duration, PRP, lens status, age, nephropathy, BMI and HbA1c; Significant p values are in bold. | | | | | | |

| **Supplementary S2. Outcome stratified by injection type** | | | |
| --- | --- | --- | --- |
| **Injection type** | **Insulin** | **OHA** | **P value** |
| **Bevacizumab** | **N=75** | **N=34** |  |
| Baseline BCVA (approxETDRS) | 62.25 (13.71) | 60.94 (13.43) | 0.642 |
| Final BCVA (approxETDRS) | 65.99 (14.55) | 62.09 (16.86) | 0.221 |
| Baseline CMT (microns) | 385.51 (101.93) | 424.41 (116.04) | 0.080 |
| Final CMT (microns) | 336.72 (79.25) | 334.76 (90.16) | 0.909 |
| **Ranibizumab** | **N=24** | **N=14** |  |
| Baseline BCVA (approxETDRS) | 67.29 (10.63) | 65.36 (12.92) | 0.620 |
| Final BCVA (approxETDRS) | 70.29 (10.44) | 69.57 (9.08) | 0.831 |
| Baseline CMT (microns) | 358.13 (58.51) | 368.86 (122.01) | 0.716 |
| Final CMT (microns) | 302.88 (47.46) | 288.07 (50.45) | 0.371 |
| Abbreviations: BCVA=best corrected visual acuity; CMT=central macular thickness; approxETDRS=approximate early treatment diabetic retinopathy study; OHA=oral hypoglycemic agent  Data are presented as means ± SD. *p-values are for a difference between insulin and OHA group. Statistical test: Independent t-test; Significant p-values are in bold. | | | |

| **Supplementary S3: Anti-VEGF treatment outcome in patients receiving insulin or OHA for DM, in participants with good final vision** | | | |
| --- | --- | --- | --- |
|  | **Insulin** | **OHA** | **P value^*^ (95% CI)** |
| **BCVA ≥70 approxETDRS** | **N=86** | **N=34** |  |
| Baseline BCVA (approxETDRS) | 68.81 (9.09) | 69.65 (6.68) | 0.629 (-4.24, 2.57) |
| Final BCVA (approxETDRS) | 75.02 (4.79) | 75.15 (5.29) | 0.901 (-2.10, 1.85) |
| BCVA change (approxETDRS) | 6.21 (8.57) | 5.50 (7.27) | 0.746 (-1.00, 4.99) |
| Baseline CMT (microns) | 372.26 (84.09) | 362.12 (80.02) | 0.547 (-23.15, 43.42) |
| Final CMT (microns) | 305.47 (54.76) | 302.53 (50.97) | 0.787 (-18.62, 24.49) |
| CMT change (microns) | -66.78 (87.80) | -59.59 (81.77) | 0.653 (-30.99, 21.00) |
| Injection number | 8.36 (2.63) | 7.79 (2.83) | 0.301 (-0.515, 1.647) |
| Abbreviations: BCVA=best corrected visual acuity; CMT=central macular thickness; CI=confidence interval; DM=diabetes mellitus; approxETDRS=approximate early treatment diabetic retinopathy study; OHA=oral hypoglycemic agent  Data are presented as means (SD) for continuous variables and percentage (%) for categorical variables. N represents the total case number in each group. *p-values are for a difference between insulin and OHA group. 95% CI is for the difference between the means of the two groups. Independent t-test/ Mann-Whitney U test for continuous variables between treatment groups; Significant p-values are in bold. | | | |
